# Therapy concepts in type 1 diabetes mellitus treatment: disease modifying versus curative approaches

**DOI:** 10.1007/s00109-024-02494-w

**Published:** 2024-10-18

**Authors:** Sigurd Lenzen, Anne Jörns

**Affiliations:** 1 Institute of Experimental Diabetes Research, Hannover Medical School, 30625 Hannover, Germany; 2 Institute of Clinical Biochemistry, Hannover Medical School, Hannover, Germany

**Keywords:** Autoimmune diseases, Type 1 diabetes mellitus, Latent autoimmune diabetes in adults, Disease modifying and curative therapies

## Abstract

For many autoimmune diseases, including type 1 diabetes mellitus (T1DM), efforts have been made to modify the disease process through pharmacotherapy. The ultimate goal must be to develop therapies with curative potential by achieving an organ without signs of parenchymal cell destruction and without signs of immune cell infiltration. In the case of the pancreas, this means regenerated and well-preserved beta cells in the islets without activated infiltrating immune cells. Recent research has opened up the prospect of successful antibody combination therapy for autoimmune diabetes with curative potential. This goal cannot be achieved with monotherapies. The requirements for the implementation of such a therapy with curative potential for the benefit of patients with T1DM and LADA (latent autoimmune diabetes in adults) are considered.

## Introduction

Insulin replacement is the established therapy of type 1 diabetes mellitus (T1DM) since hundred years [1]. Anti-inflammatory therapy is a cornerstone in the treatment of many other autoimmune diseases, but not in T1DM. In recent years, the administration of therapeutic antibodies has become a new therapeutic option for the treatment of the around hundred autoimmune diseases. Typically, the therapy principle is a monotherapy with a single antibody, often in combination with other immunosuppressive drugs [2, 3]. Prominent examples include rheumatoid arthritis [4], inflammatory bowel diseases [5], and psoriasis as well as psoriatic arthritis [6, 7]. Such therapies are classified as “disease modifying” [3].

In rheumatoid arthritis, “symptom free remission” can be achieved in a subgroup of patients if diagnosed at an early stage [8]. However, so far none of the established therapy strategies for any of the autoimmune diseases has the potential to cure a disease. This is also true for children and young adults with T1DM as well as in older patients, who typically have a form of the disease known as LADA (latent autoimmune diabetes in adults), which is autoimmune in nature with an activated immune cell infiltrate in the pancreatic islets [9], but with differences in the disease progression [9].

Combination therapies are well established and are the gold standard in the treatment of many other diseases. A key aim of many combination therapies is to minimise the risk of drug resistance development. This is the case, for example, with combination therapies for infectious diseases and cancer. Combination therapies markedly increase the likelihood of curing the disease. An additional advantage is the possibility of reducing the doses of the different drugs in the combination and at the same time increasing the effectiveness of the therapy, by taking advantage of the synergistic and additive actions of the drugs.

### The ultimate type 1 diabetes mellitus therapy goal

T1DM is an autoimmune disease, with potentially serious late complications and a significant risk of death due to hypoglycaemic or hyperglycaemic episodes [10]. In addition, the socioeconomic burden of T1DM is enormous, with a recent estimate of up to one trillion dollars in lifetime costs in the USA alone [11]. It is therefore surprising that the pressure to develop antibody-based therapies with curative potential is not greater in the medical and lay public [12–14]; although, there is a broad consensus in the diabetes community that antibody-based combination therapies are needed to achieve this goal [15–21].

At the experimental level in the animal model of human type 1 diabetes (the IDDM rat) [22], the curative therapy success with antibody combinations is based on two elements. Since autoimmune diseases are mediated by proinflammatory cytokines (especially TNF-α and IL-1β) [9, 23], which are responsible for dysfunction of the respective target cells effective therapeutic regimes require treatment with a TNF-α antibody [24]. In a recent clinical trial after the onset of T1DM, monotherapy with human anti-TNF-α showed a mild effect on preserved C-peptide concentration as a surrogate for insulin secretion after a mixed meal [25]. This is consistent with previous findings of the beneficial metabolic effect of anti-TNF-α in the treatment of another autoimmune disease in patients who developed additionally T1DM [26, 27]. In contrast, the human anti-IL-1β Xoma 052 (gevokizumab) did not provide C-peptide preservation in the T1DM setting.

Without a complete elimination of the activated immune cell infiltrate, especially cytotoxic CD8 T cells, but also CD4 T cells and macrophages [9, 23], it is not possible to stop the gradual destructive apoptotic process of the remaining beta cells. Nor is it possible to switch to an effective beta cell proliferation with the aim of increasing the beta cell mass to a volume that does not require insulin supplementation. All other therapies, monotherapies as well as combination therapies, that do not have this potential, are not curative as shown in the IDDM rat model of human T1DM [24, 28, 29].

### Prerequisites for successful combination therapies of type 1 diabetes mellitus

**Fig. 1:**
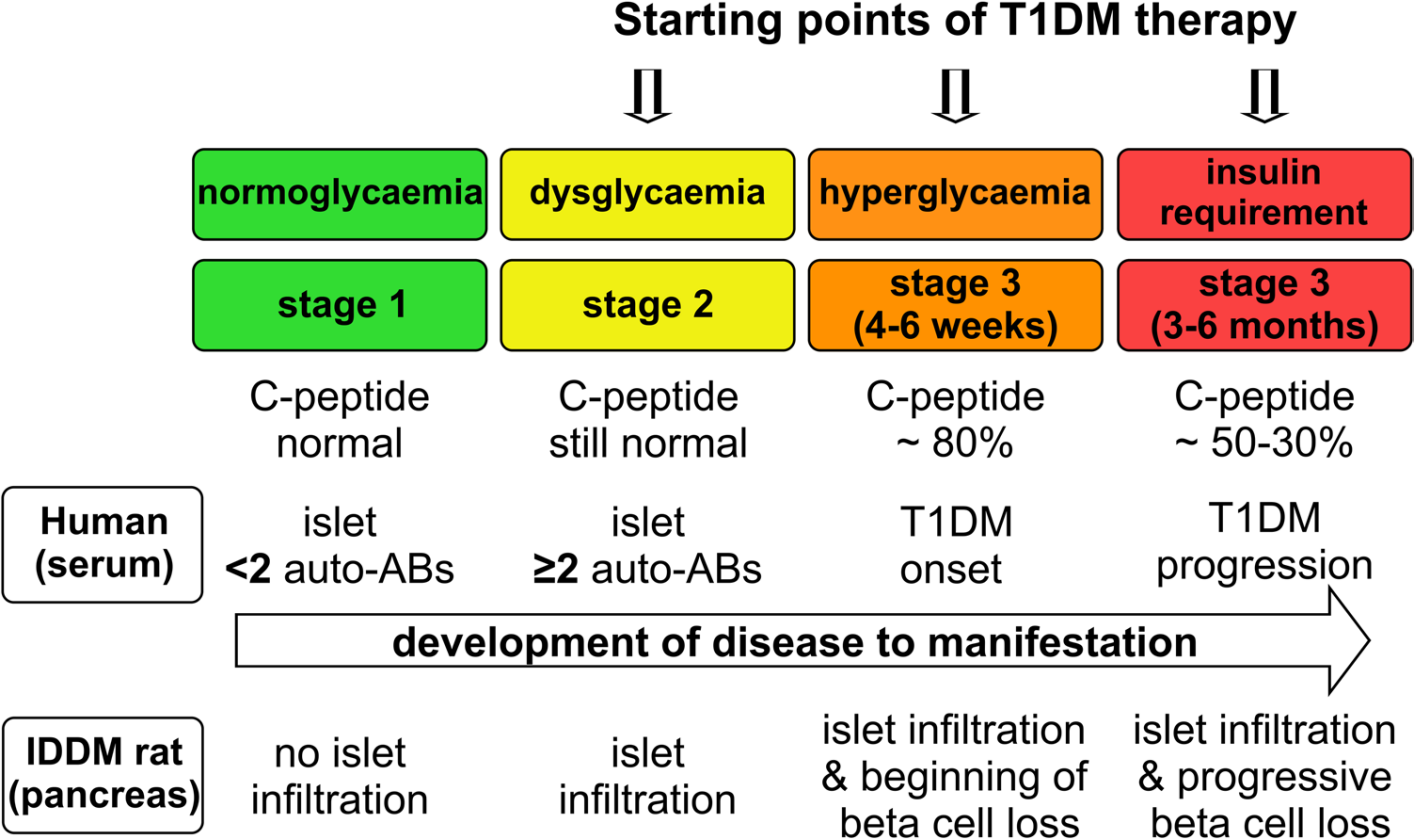
Starting points of type 1 diabetes mellitus therapy. The metabolic stages before and after diabetes manifestation with C-peptide concentrations and islet autoantibodies (ABs) in the human situation and the changes in the immune cell infiltration (in particular CD4 and CD8 T cells and macrophages) in the islets of the pancreas in the IDDM rat model of human T1DM are depicted

There exist two humanised anti-CD3 antibodies, otelixizumab (GSK, Stevenage, UK) [30] and teplizumab (Provention Bio, Oldwick, N.J., USA) [31, 32]. At low doses needed in combination therapies, they have been shown to be safe in clinical trials [32, 33]. More recently (in November 2022), the FDA has approved teplizumab for a first circumscribed indication for disease modification in T1DM. T1DM is a chronic inflammatory process in the pancreas with different stages before and after disease onset. In the human situation, changes in circulating metabolic markers such as glucose and C-peptide concentrations as well as islet auto-antibodies have been shown to correlate very well with changes in pancreatic islets in the rat model of human T1DM in terms of beta cell loss and levels of immune cell infiltration (Fig. 1).

This explains why such therapies for patients with T1DM, e.g. with an anti-CD3 monotherapy [34–40], through insulin immunisation [12, 41, 42] or other autoantigen treatments [12, 41], have been unsuccessful in reversing the diabetic metabolic state in patients with stage 3 of T1DM. A wide range of different immunotherapies have been evaluated [43–46]. Efforts to achieve this goal by starting therapy early after the development of islet autoantibodies (stage 2) and before the manifestation of diabetic hyperglycaemia have not been successful. Even clinical trials with the most effective monotherapies have failed to demonstrate a curative potential in recent onset T1DM [46, 47]. It is therefore logical that anti-CD3 therapy is referred to “disease modifying therapy” by the Trial Network scientists [48]. In a recent clinical trial (phase Ib/IIa), anti-CD3 therapy was combined with AG019, *Lactococcus lactis* bacteria delivering proinsulin and IL-10, and showed a slight increase in C-peptide concentrations 6 months after treatment. Even this combination was not effective over a longer period [49].

The combination antibody therapy that has been shown to reverse islet immune cell infiltration and diabetic hyperglycaemia in the IDDM rat model of human T1DM is a combination of anti-TCR/CD3 with anti-TNF-α [24]. This combination can lead to a cure of T1DM without the need for insulin supplementation by complete abolition of immune cell infiltration in the islets together with beta cell preservation and subsequent proliferation of beta cells through different regeneration mechanisms [24, 29]. The final therapeutic success is characterised by well preserved and regenerated beta cells in the islets with no signs of immune cell activation.

Combination therapy blocks the beta cell destructive potential, mediated by the release of beta cell toxic T cell mediators and proinflammatory cytokines. It is successful when an intervention to stop of the apoptotic process is initiated before more than 2/3 of the normal healthy beta cell mass is lost [24, 29, 50]. However, this also requires diagnosis of T1DM at an early stage, when a large residual beta cell mass is still present.

### Possible therapy regimens

What would a treatment schedule for a combination therapy look like? In combination therapy, both the anti-CD3 antibody (Tzield®) and the anti-TNF-α antibody (Humira®) should definitely be administered by intravenous infusion. We know from animal studies that this is more effective than subcutaneous administration. Clinical monotherapy studies with the anti-CD3 antibody teplizumab have used two different dosing regimens.

When combining anti-CD3 with anti-TNF-α, the two antibodies should not be administered at the same time to avoid the risk of interaction between the two antibodies during infusion. Separate application, e.g. in the morning and afternoon, would be possible. The daily doses to be administered should be those that are approved by the regulatory authorities and indicated in the information leaflets by the pharmaceutical companies for the respective application forms, in the case of anti-CD3 for treatment of T1DM and in the case of anti-TNF-α for the treatment of other autoimmune diseases such as rheumatoid arthritis. In addition to same-day application, the following variations in the antibody administration in the combination therapy could be considered:

I. Start of the 14-day anti-TNF-α therapy, 3 days before the start of the 14-day anti-CD3 therapy (17 days in total).
II. In a sequential therapy regimen, anti-TNF-α would be given for 7 days, followed by 14 days of anti-CD3 and a further 7 days of anti-TNF-α administration (28 days in total).
III. A viable strategy could also comprise two consecutive monotherapies, an initial 14 day anti-CD3 monotherapy followed by a consecutive 14 day anti-TNF-α monotherapy, which has been shown to be safe and effective in the IDDM rat model of human T1DM [50].

A second course of treatment with teplizumab should not be considered. However, after completion of the combination therapy, a follow-up therapy with a low dose of the sphingosine-1-phosphate analogue fingolimod (Gilenia®) could be worth considering to retain activated immune cells in the peripheral lymph nodes (especially in the pancreas draining lymph nodes to prevent re-infiltration of the pancreatic islets) [28]. The aim of all therapeutic efforts with a curative goal must be sustainability, ideally with a permanent freedom from immune cell infiltration of the pancreatic islets for many years [29].

Such a combination therapy with the two therapeutic antibodies is particularly interesting for children and adolescents who suffer from rheumatoid arthritis and subsequently develop T1DM. The same applies to the treatment of T1DM along with other autoimmune diseases.

Treatment with the anti-CD3 antibody teplizumab at the dose administered in monotherapy in clinical trials is associated with transient lymphopenia that begins during therapy and persists for the first 3 weeks after therapy. Lymphocyte counts then return to normal [51]. At lower doses, which are possible in combination therapies and have been shown to be safe in clinical trials [32, 33], this undesirable effect should be even less pronounced.

### Therapy success control

In the clinical trials with monotherapies, i.e. anti-CD3, the therapeutic goal, a retardation of the disease process [48] is documented by a slowing of the decline of the C-peptide concentration as an indicator for a retardation of the beta cell destruction process and a resulting delayed development of diabetic hyperglycaemia along with a reduction of insulin requirements [34].

To determine the success of combination therapies with curative potential, it would be desirable to obtain information on the status of islet immune cell infiltration as it is possible by morphological analyses in pancreatic biopsies in animal models of human T1DM during the course of the disease process [24]. As long as this is not possible in patients, for example due to lack of reliable imaging techniques, preventive therapies can only be started after the onset of the islet immune cell infiltration. Any therapy with curative potential must therefore reverse islet immune cell infiltration.

Ideally, T1DM therapy success should be maintained for lifetime, at least for decades in children and young adults with T1DM and for many years in patients with LADA. However, such a long-term therapy success cannot be predicted at present. For this reason, the patient should be monitored continuously after antibody treatment has stopped to detect any deterioration in the metabolic state at an early stage.

In the case of a “disease modifying monotherapy” with the anti-CD3 antibody, the delay in disease progression can be determined by a prevention of plasma C-peptide decline for a few years. In contrast, in the case of an antibody combination therapy with curative potential, the monitoring of therapy success must focus on a sustained increase in the plasma C-peptide concentration towards the normal range. Plasma C-peptide levels must be controlled regularly to detect early undesirable decreases of C-peptide levels, which would be indicative of a relapse of islet immune cell infiltration. There is a need for further identification of reliable metabolic biomarkers [45], none of which have yet achieved the status of an established biomarker in clinical diabetology for reliable monitoring of pancreatic islet immune cell infiltration.

It remains to be seen whether a sustained therapeutic success can be maintained by preventing re-infiltration of the pancreatic islets with activated immune cells producing cytotoxic proinflammatory cytokines. An attractive option to achieve this goal might be the administration of a low dose of fingolimod (FTY720), which can prevent such a re-infiltration [52].

## Conclusion

In the future, there will be two different new therapy options for patients to choose from. Either a so-called “disease modifying monotherapy” with anti-CD3 (i.e. teplizumab), which is in fact a “disease progression retarding therapy” with the ability to delay disease progression for a few years [39, 53, 54], or an antibody combination therapy with the prospect of a long-term curative effect, consisting of an administration of anti-CD3 plus anti-TNF-α [24, 50].

We know from studies in animal models of human T1DM [24, 50], which combinations we need to choose in order to successfully exploit the curative potential of antibody-based combination therapies, which are more effective even at lower doses than monotherapies and at the same time have fewer side effects.
